# Supplementary material for: (E)-1-(Furan-2-yl)-(substituted phenyl)prop-2-en-1-one Derivatives as Tyrosinase Inhibitors and Melanogenesis Inhibition: An In Vitro and In Silico Study
Source: Molecules. 2020 Nov 21;25(22):5460. doi: 10.3390/molecules25225460 (PMC7700175; doi:10.3390/molecules25225460)
Supplement: Supplementary file 1 [file molecules-25-05460-s001.pdf]

Supporting Information

For

(*E*)-1-(Furan-2-yl)-(substituted phenyl)prop-2-en-1 one derivatives as tyrosinase inhibitors and melanogenesis inhibition: An *in vitro* and *in silico* study

Hee Jin Jung[1], Sang Gyun Noh[1], Il Young Ryu[1], Chaeun Park[1], Ji Young Lee[1], Pusoon Chun[2], Hyung Ryong Moon[1,*], Hae Young Chung[1,*]

[1]*College of Pharmacy, Pusan National University, Busan 46241, Republic of Korea.* [2]*College of Pharmacy and Inje Institute of Pharmaceutical Sciences and Research, Inje University, Gimhae, Gyeongnam 50834, South Korea*

Contents

Fig. S1. ^{1}H NMR spectrum of compound **1**……………………………………………….................4

Fig. S2. ^{13}C NMR spectrum of compound **1**…………………………………………………….…4

Fig. S3. ESI-MS spectrum of compound **1**……………………………………………………………5

Fig. S4. ^{1}H NMR spectrum of compound **2**……………………………………………….................6

Fig. S5. ^{13}C NMR spectrum of compound **2**…………………………………………………….….6

Fig. S6. ESI-MS spectrum of compound **2**……………………………………………………………7

Fig. S7. ^{1}H NMR spectrum of compound **3**……………………………………………….................8

Fig. S8. ^{13}C NMR spectrum of compound **3**………………………………………………………8

Fig. S9. ESI-MS spectrum of compound **3**……………………………………………………………9

Fig. S10. ^{1}H NMR spectrum of compound **4**…………………………………………….............10

Fig. S11. ^{13}C NMR spectrum of compound **4**……………………………………………….…10

Fig. S12. ESI-MS spectrum of compound **4**………………………………………………….…11

Fig. S13. ^{1}H NMR spectrum of compound **5**…………………………………………….............12

Fig. S14. ^{13}C NMR spectrum of compound **5**………………………………………………….12

Fig. S15. ESI-MS spectrum of compound **5**……………………………………...…………13

Fig. S16. ^{1}H NMR spectrum of compound **6**…………………………………………….............14

Fig. S17. ^{13}C NMR spectrum of compound **6**……………………………………………….…14

Fig. S18. ESI-MS spectrum of compound **6**……………………………………………………15

Fig. S19. ^{1}H NMR spectrum of compound **7**…………………………………………….............16

Fig. S20. ^{13}C NMR spectrum of compound **7**………………………………………………….16

Fig. S21. ESI-MS spectrum of compound **7**……………………………………………………17

Fig. S22. ^{1}H NMR spectrum of compound **8**…………………………………………….............18

Fig. S23. ^{13}C NMR spectrum of compound **8**…………………………………………………..18

Fig. S24. ESI-MS spectrum of compound **8**……………………………………………………19

Fig. S25. ^{1}H NMR spectrum of compound **9**…………………………………………...............20

Fig. S26. ^{13}C NMR spectrum of compound **9**………………………………………………..20

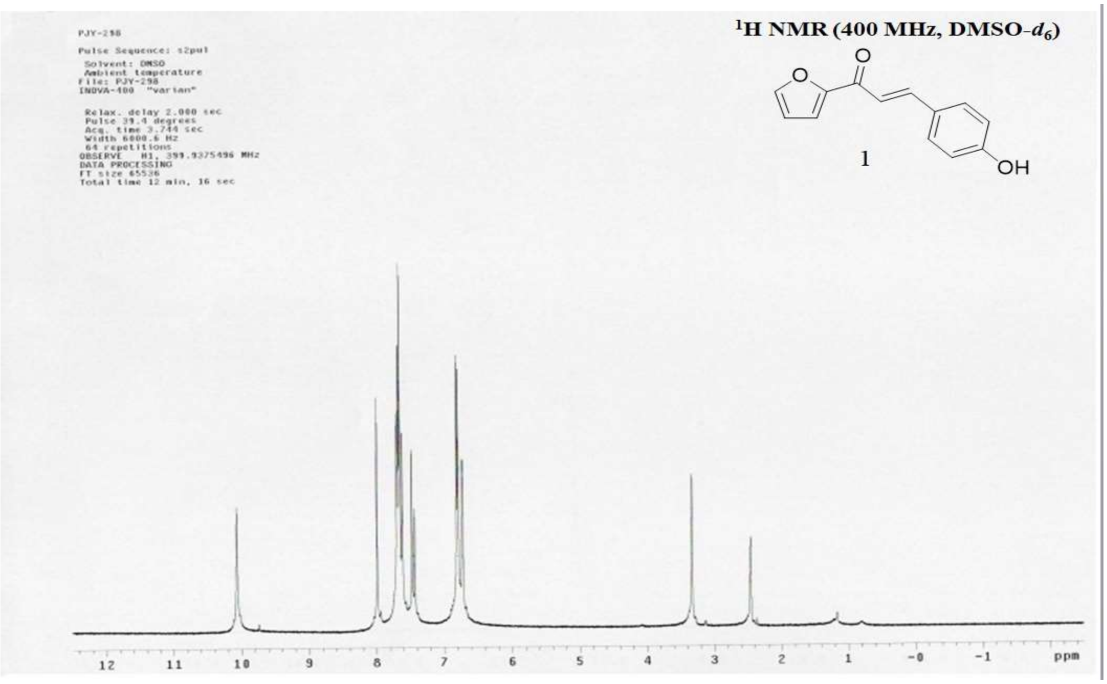

Fig. S1. ^{1}H-NMR spectrum of **1**.

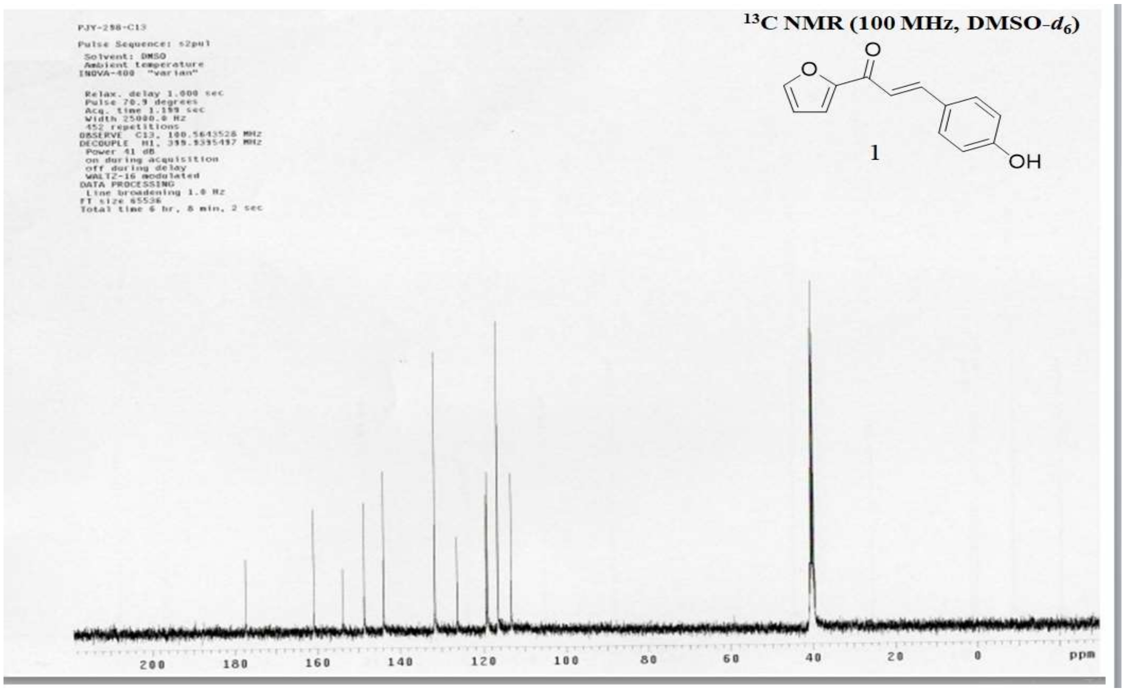

Fig. S2. ^{13}C-NMR spectrum of **1**.

HRMS (ESI+) m/z $C_{13}H_{11}O_3$ $(M+H)^+$ calcd 215.0703, obsd 215.0701.

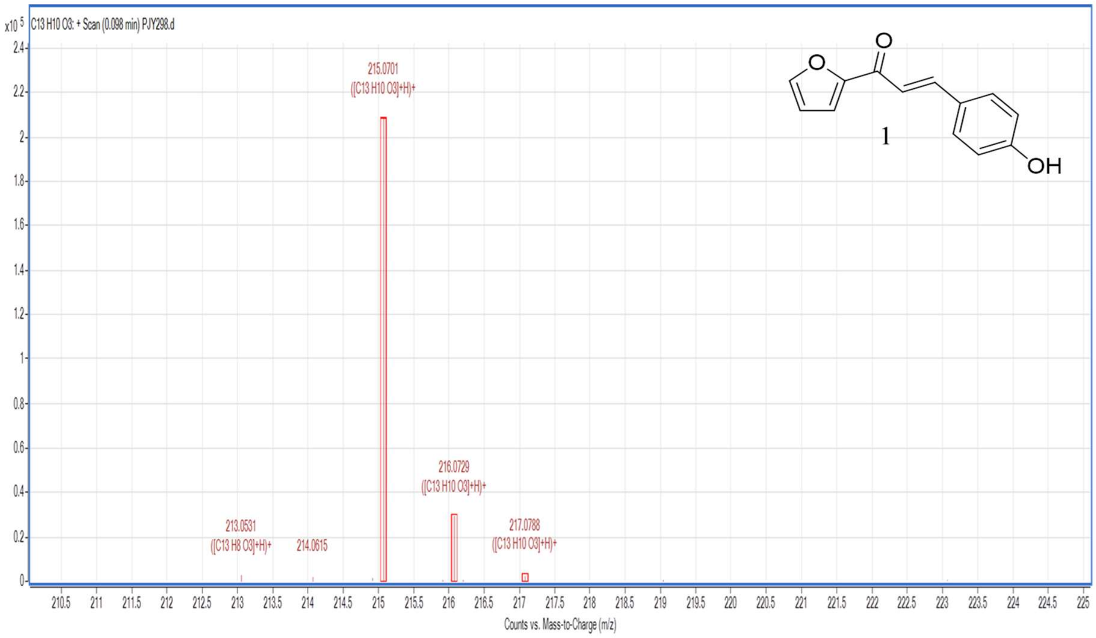

Fig. S3. ESI-MS spectrum of **1**.

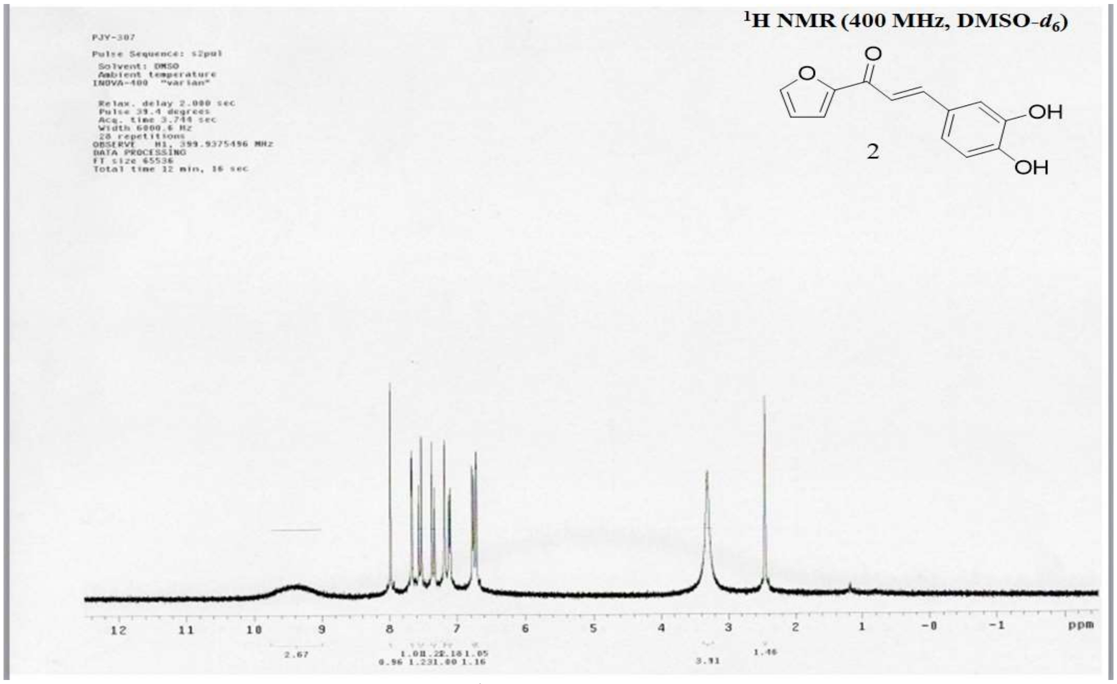

Fig. S4. ^{1}H-NMR spectrum of **2**.

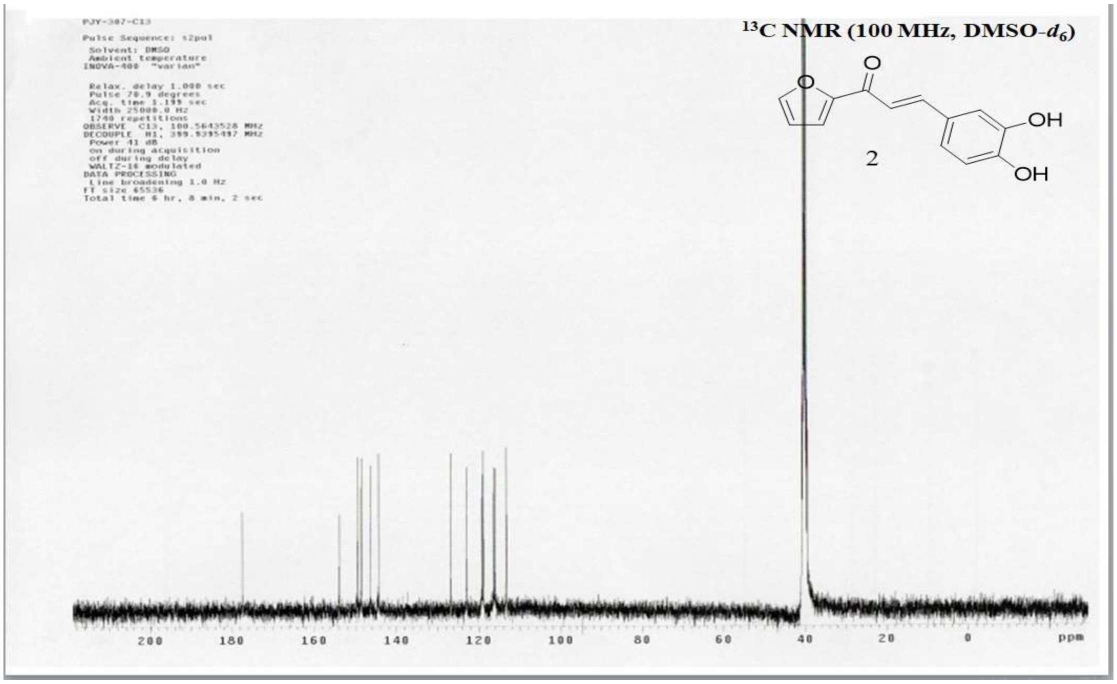

Fig. S5. ^{13}C-NMR spectrum of **2**.

HRMS (ESI+) *m/z* $C_{13}H_{11}O_4$ $(M+H)^+$ calcd 231.0652, obsd 231.0643.

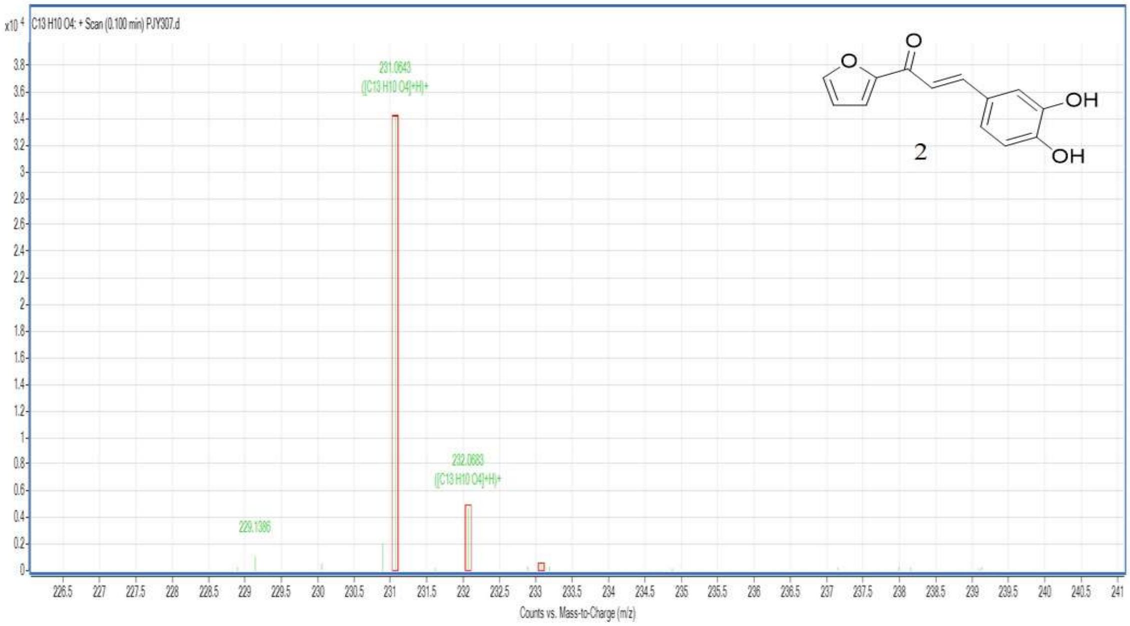

Fig. S6. ESI-MS spectrum of **2**.

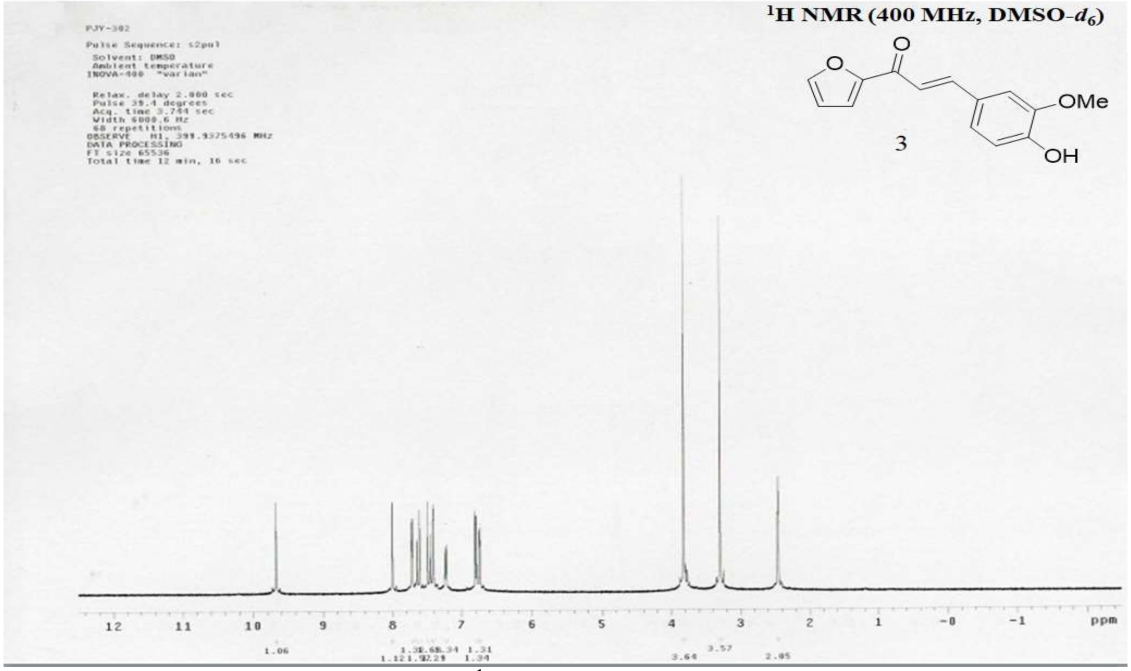

Fig. S7. ^{1}H-NMR spectrum of **3**.

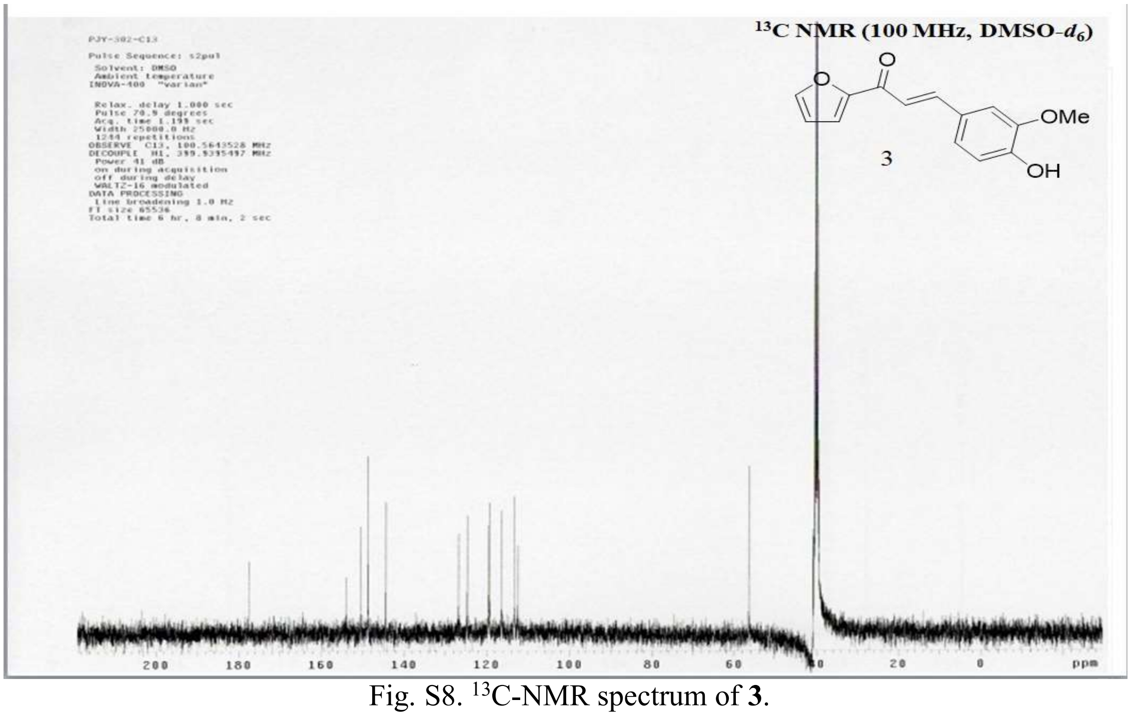

Fig. S8. ^{13}C-NMR spectrum of **3**.

HRMS (ESI+) *m/z* $C_{14}H_{13}O_4$ $(M+H)^+$ calcd 245.0808, obsd 245.0806.

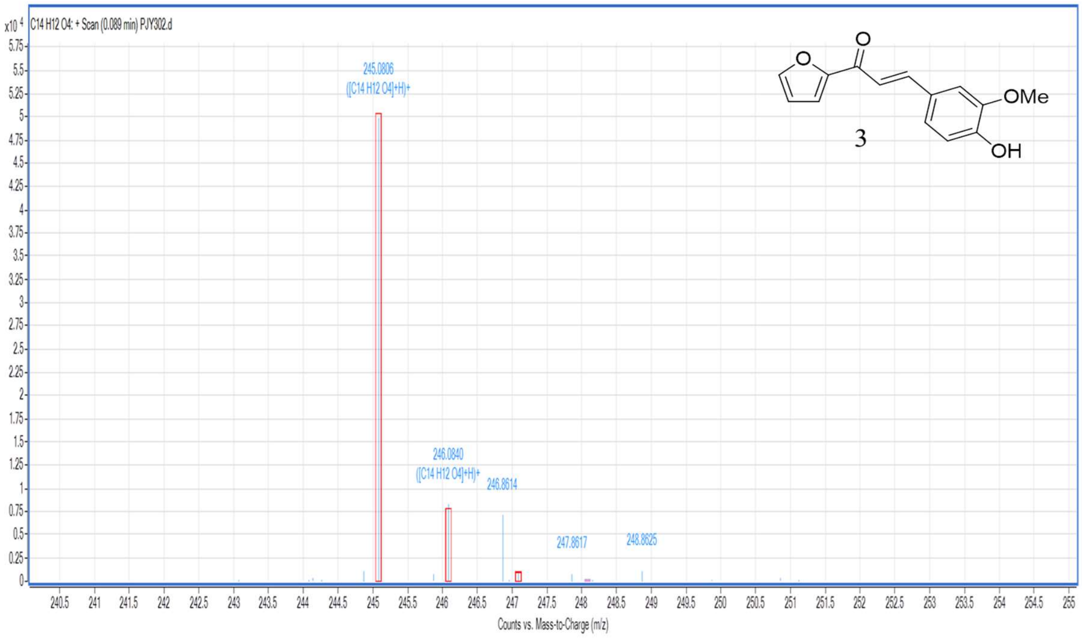

Fig. S9. ESI-MS spectrum of **3**.

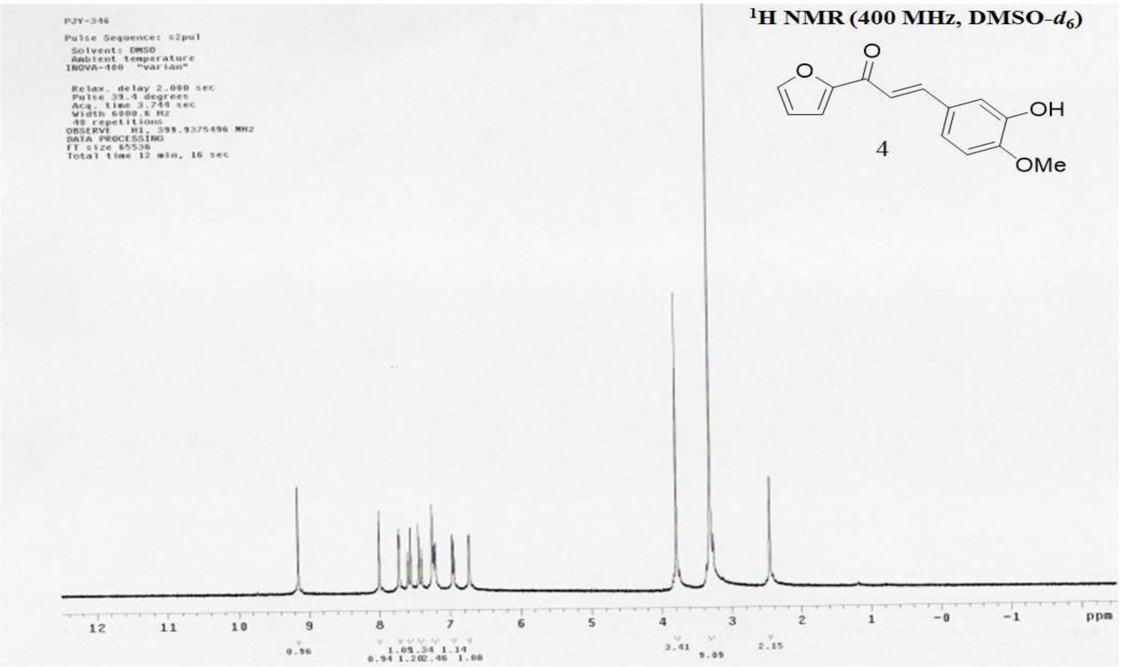

Fig. S10. ^{1}H-NMR spectrum of **4**.

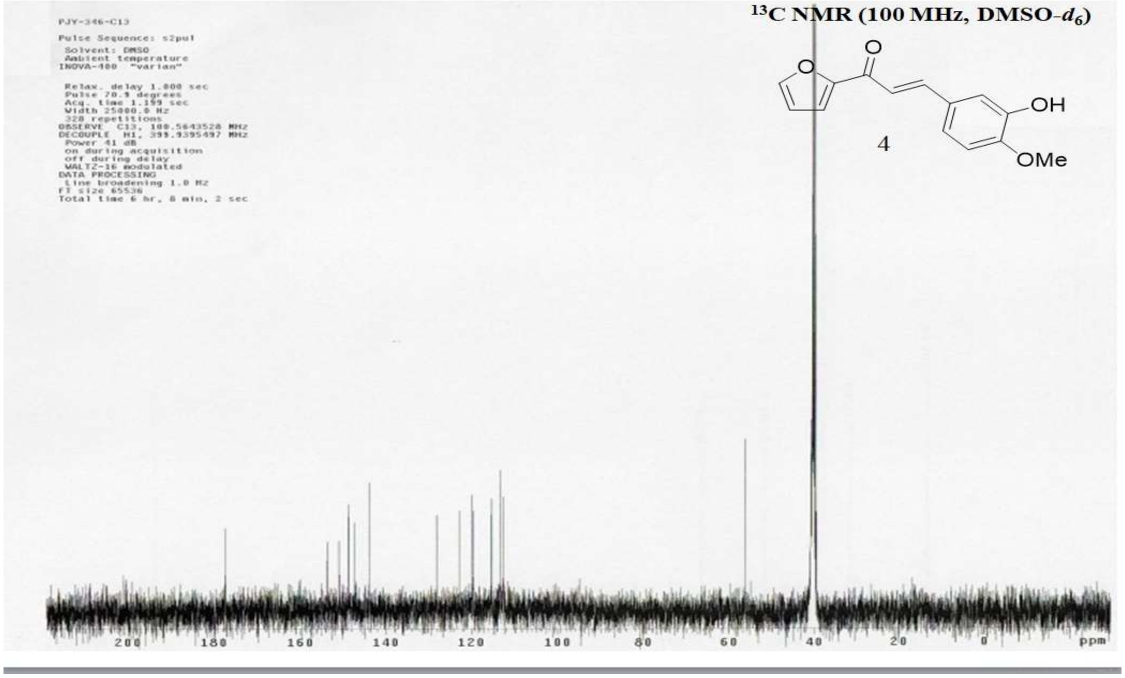

Fig. S11. ^{13}C-NMR spectrum of **4**.

HRMS (ESI+) m/z $C_{14}H_{13}O_4$ $(M+H)^+$ calcd 245.0808, obsd 245.0802.

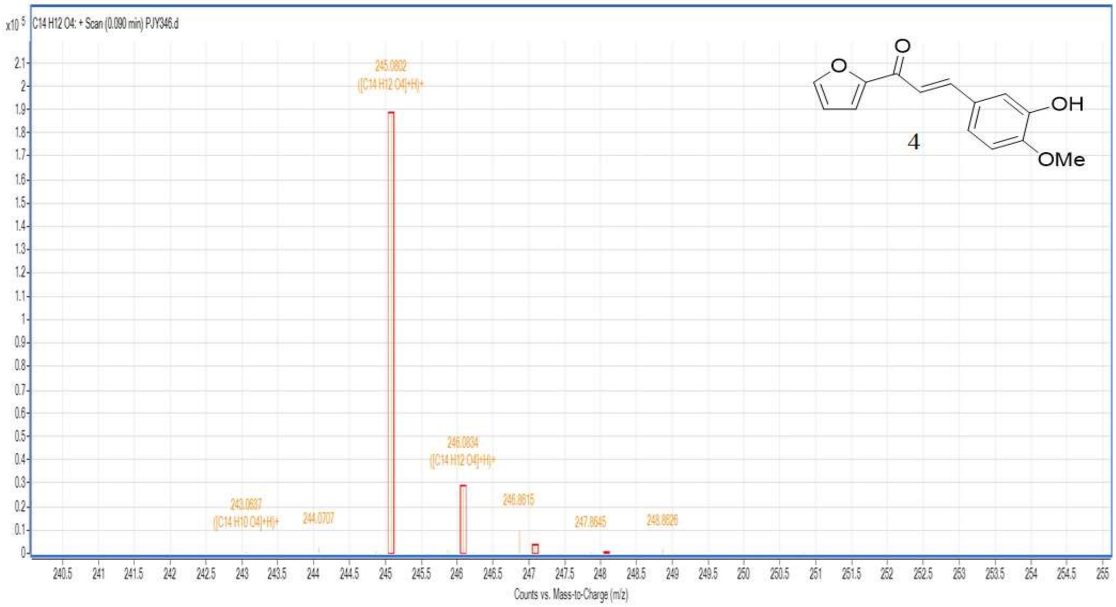

Fig. S12. ESI-MS spectrum of **4**.

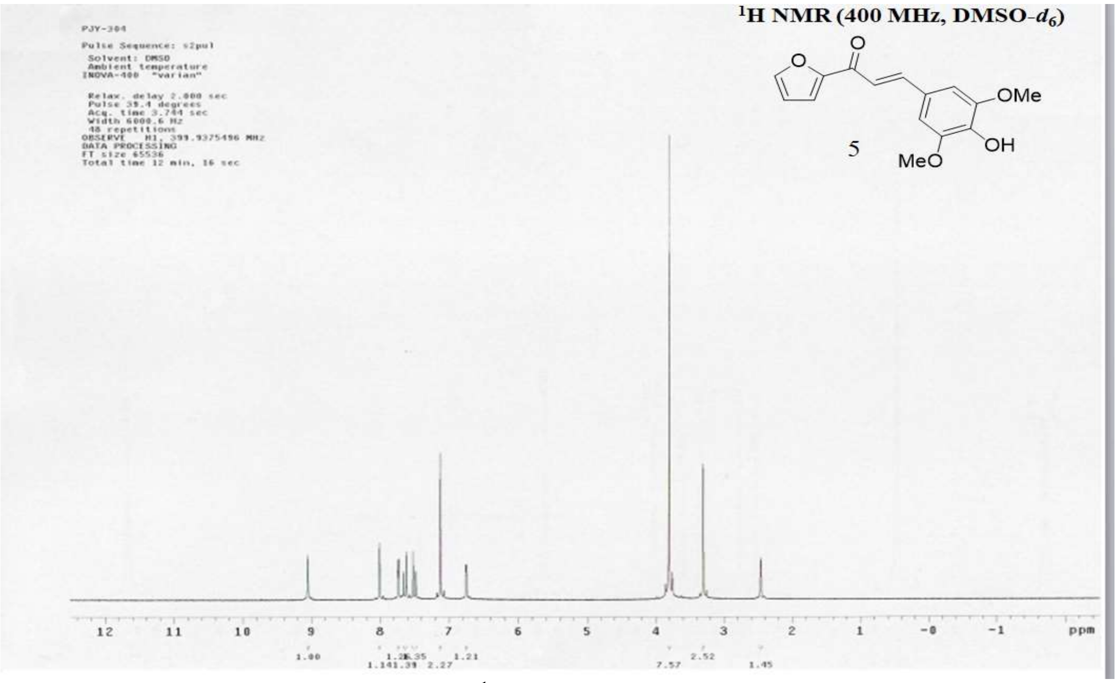

Fig. S13. ^{1}H-NMR spectrum of **5**.

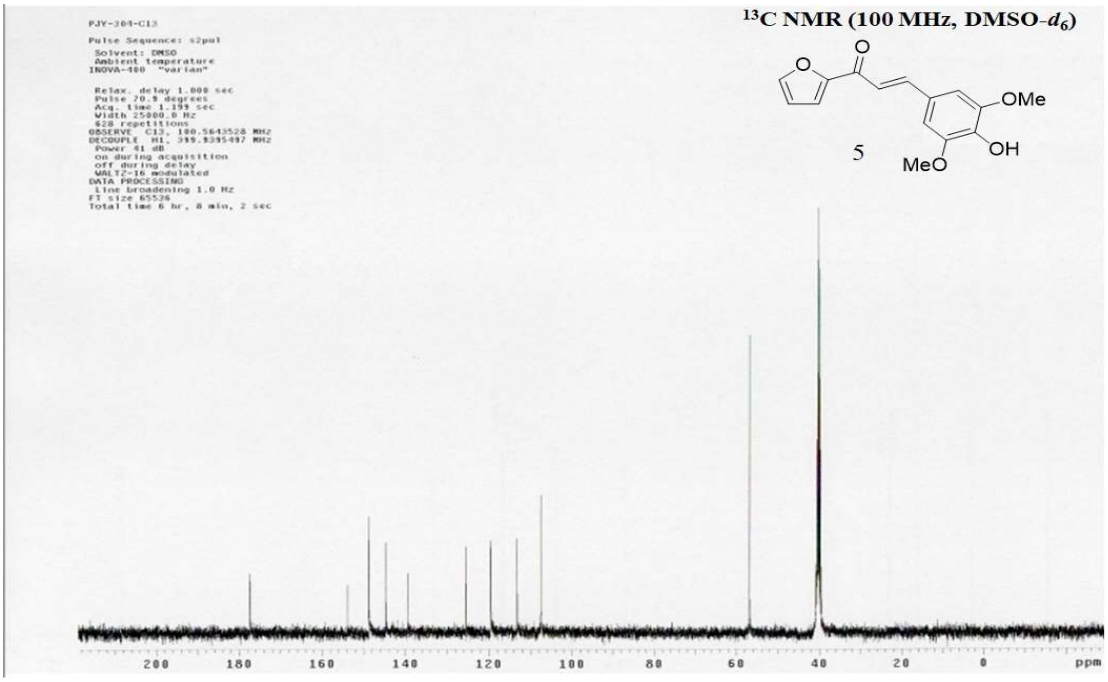

Fig. S14. ^{13}C-NMR spectrum of **5**.

HRMS (ESI+) *m/z* $C_{15}H_{15}O_5$ $(M+H)^+$ calcd 275.0914, obsd 275.0906.

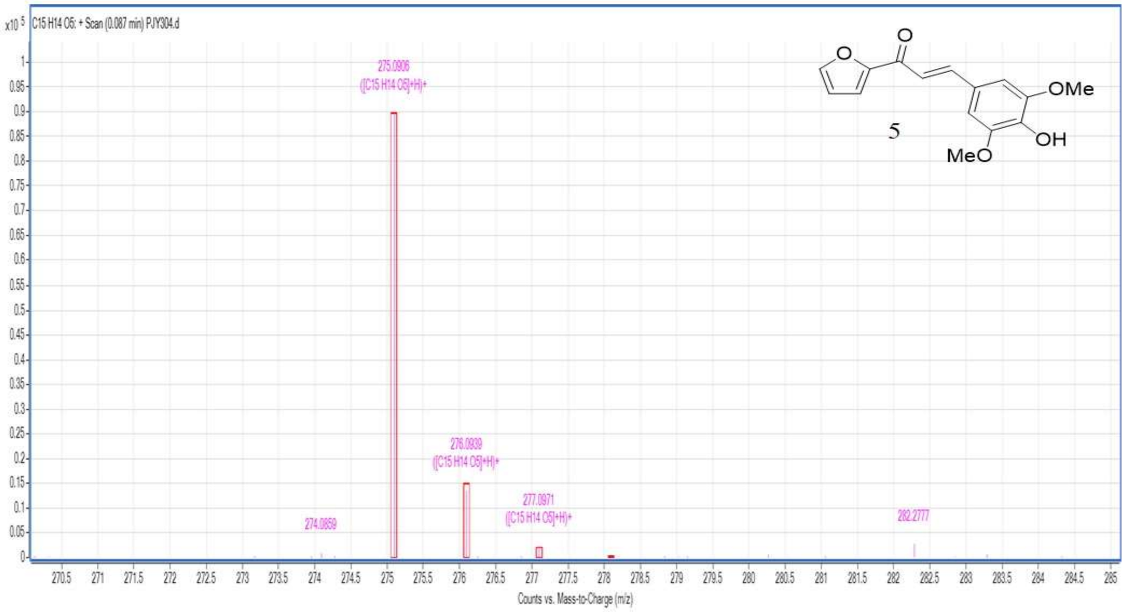

Fig. S15. ESI-MS spectrum of **5**.

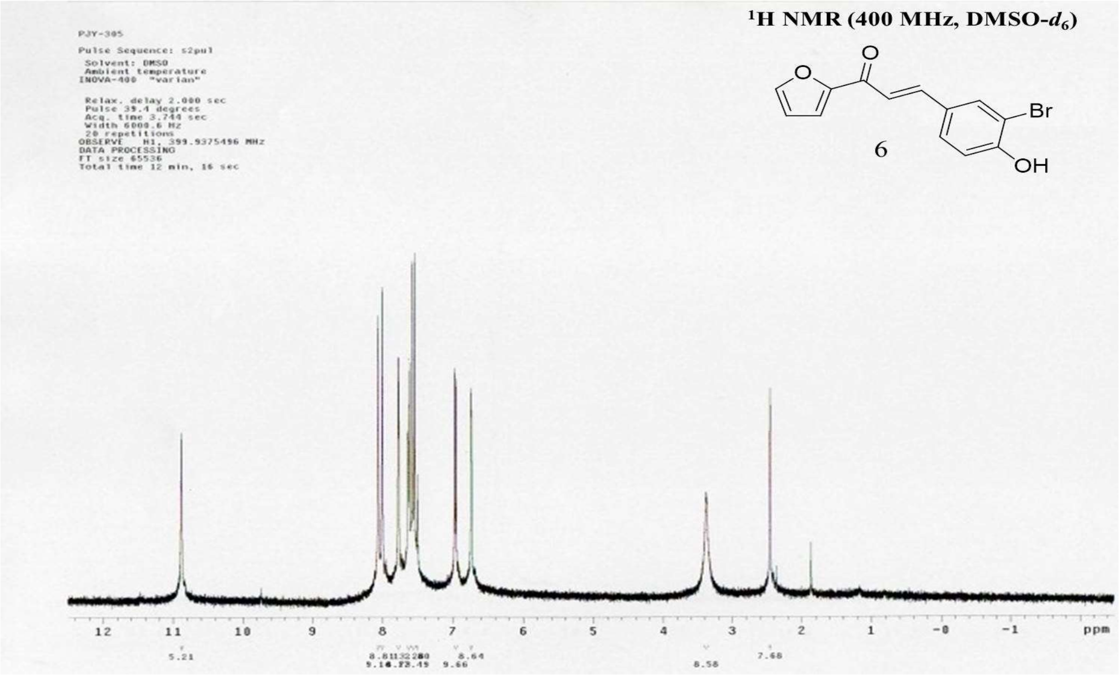

Fig. S16. ^{1}H-NMR spectrum of **6**.

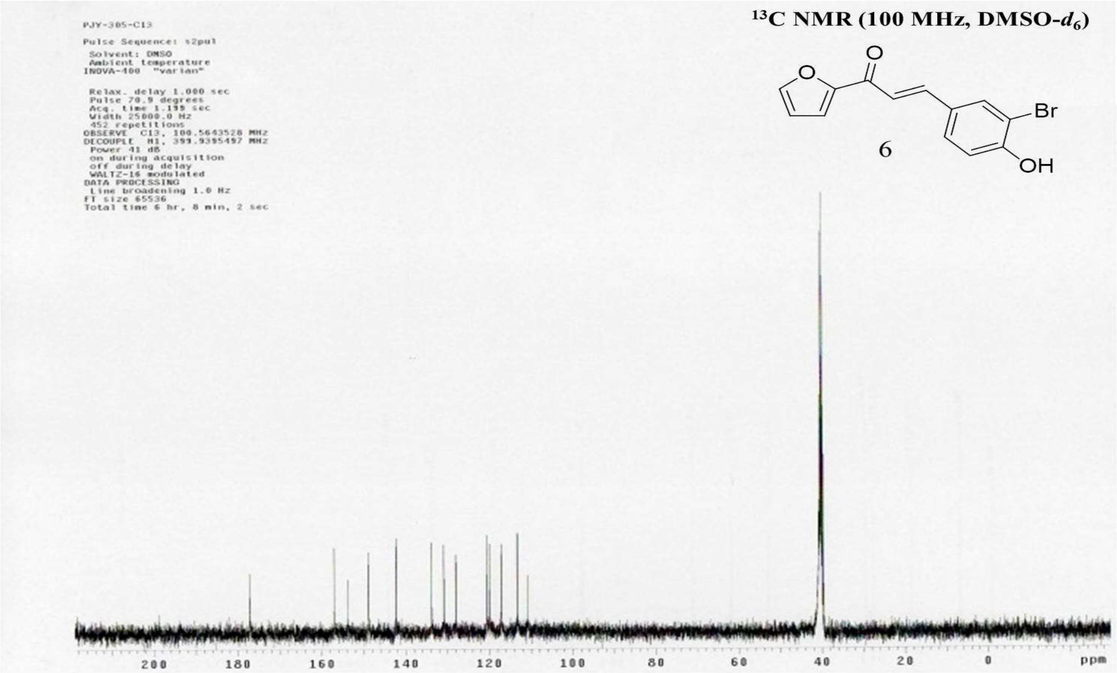

Fig. S17. ^{13}C-NMR spectrum of **6**.

HRMS (ESI+) *m/z* $C_{13}H_{10}BrO_3$ $(M+H)^+$ calcd 292.9808, obsd 292.9802, $C_{13}H_{10}BrO_3$ $(M+2+H)^+$ calcd 294.9789, obsd 294.9787.

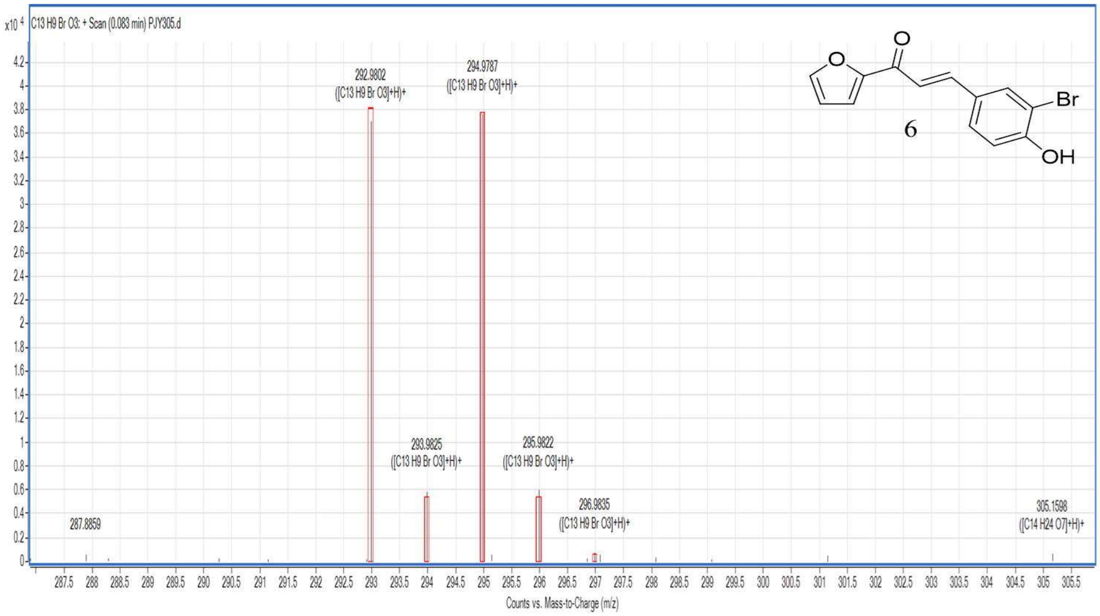

Fig. S18. ESI-MS spectrum of **6**.

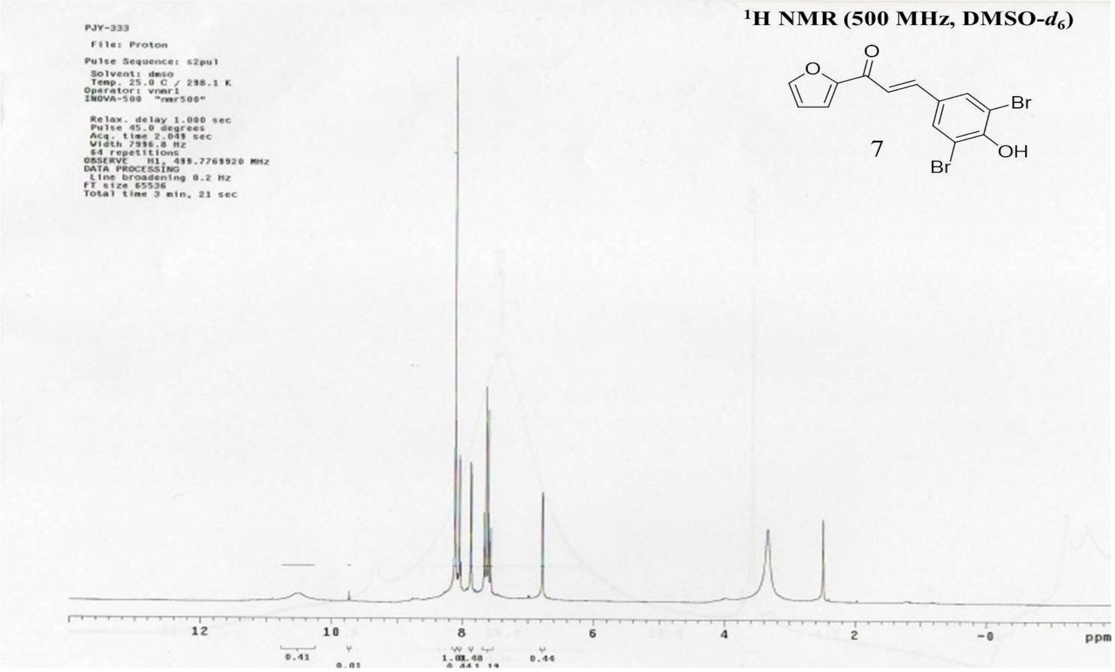

Fig. S19. ^{1}H-NMR spectrum of **7**.

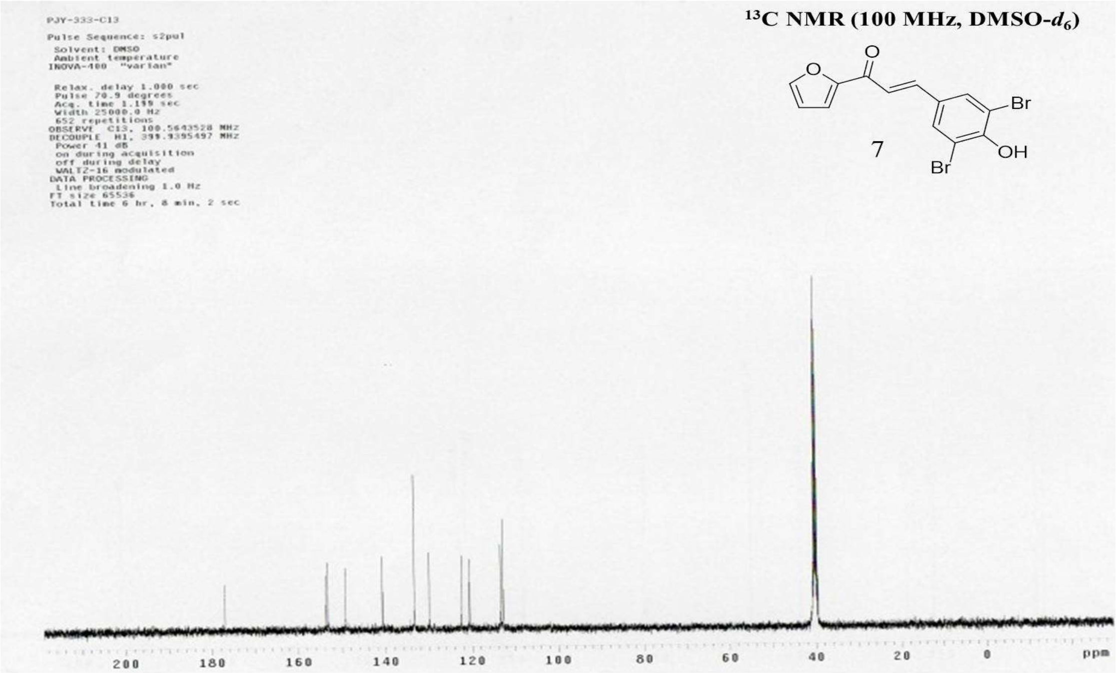

Fig. S20. ^{13}C-NMR spectrum of **7**.

HRMS (ESI+) m/z $C_{13}H_9Br_2O_3$ $(M+H)^+$ calcd 370.8913, obsd 370.8900, $C_{13}H_9Br_2O_3$ $(M+2+H)^+$ calcd 372.8893, obsd 372.8880, $C_{13}H_9Br_2O_3$ $(M+4+H)^+$ calcd 374.8874, obsd 374.8861.

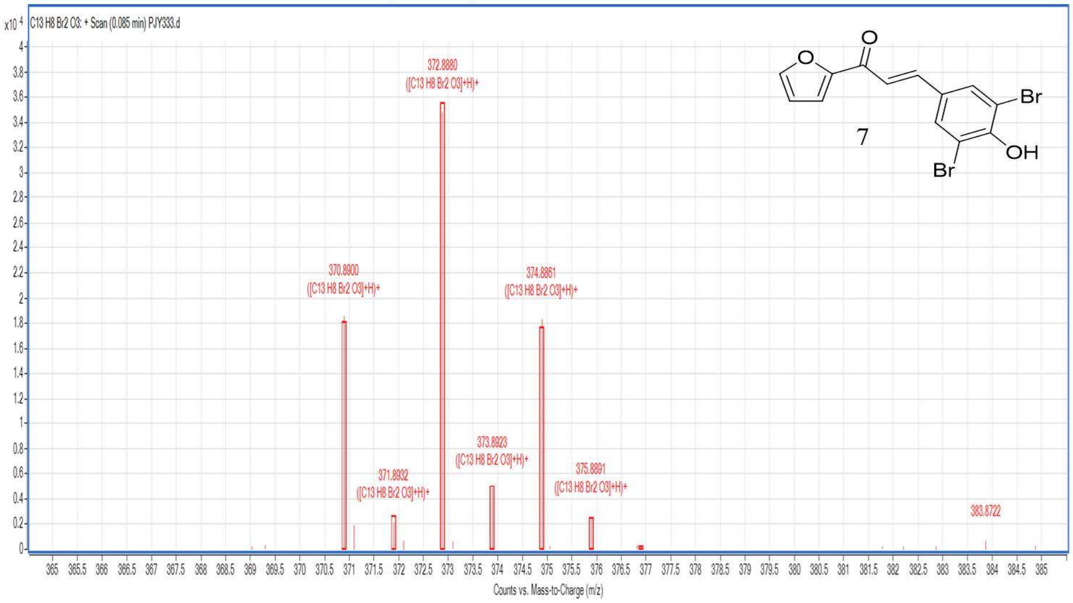

Fig. S21. ESI-MS spectrum of **7**.

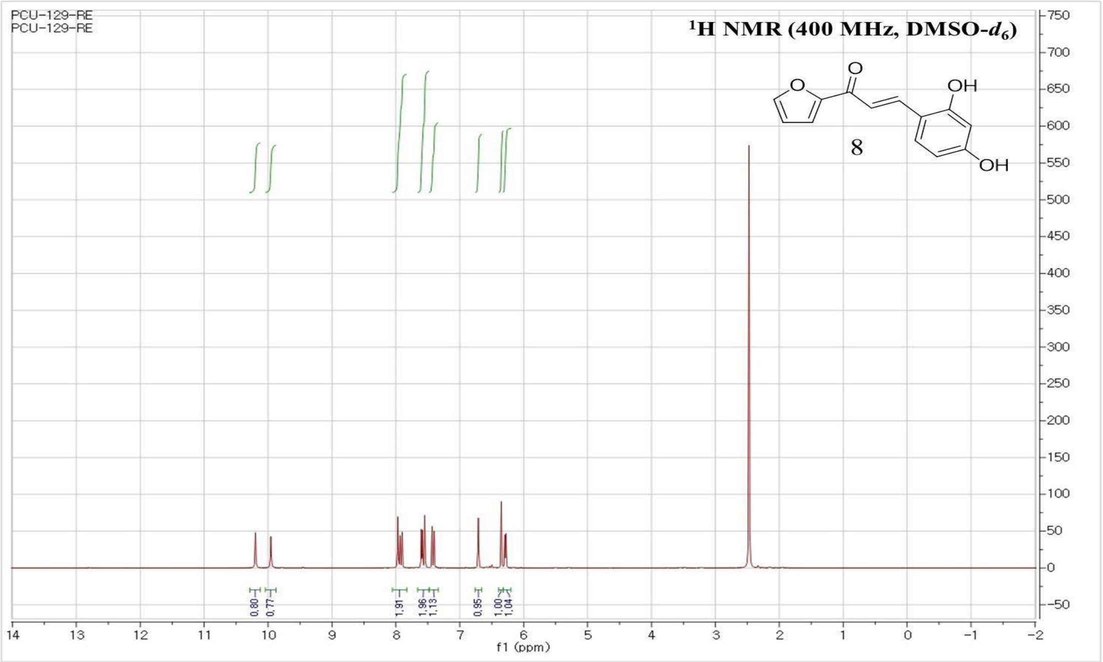

Fig. S22. ^{1}H-NMR spectrum of **8**.

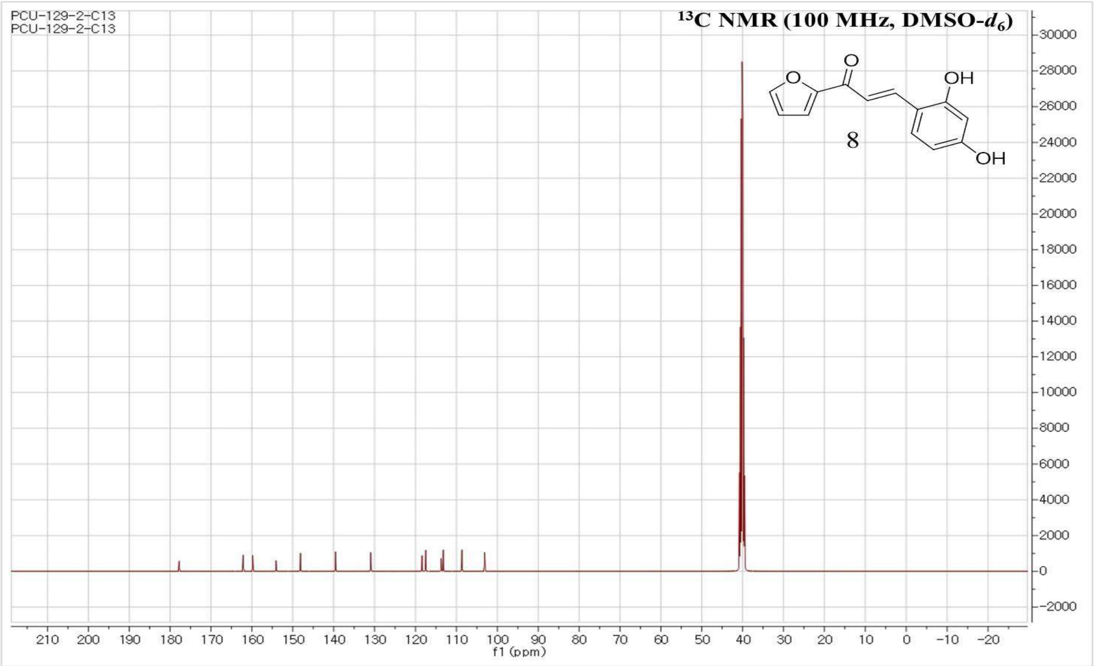

Fig. S23. ^{13}C-NMR spectrum of **8**.

LRMS (ESI-) *m/z* $C_{13}H_{10}O_4$ $(M-H)^-$ calcd 230.06, obsd 299.08.

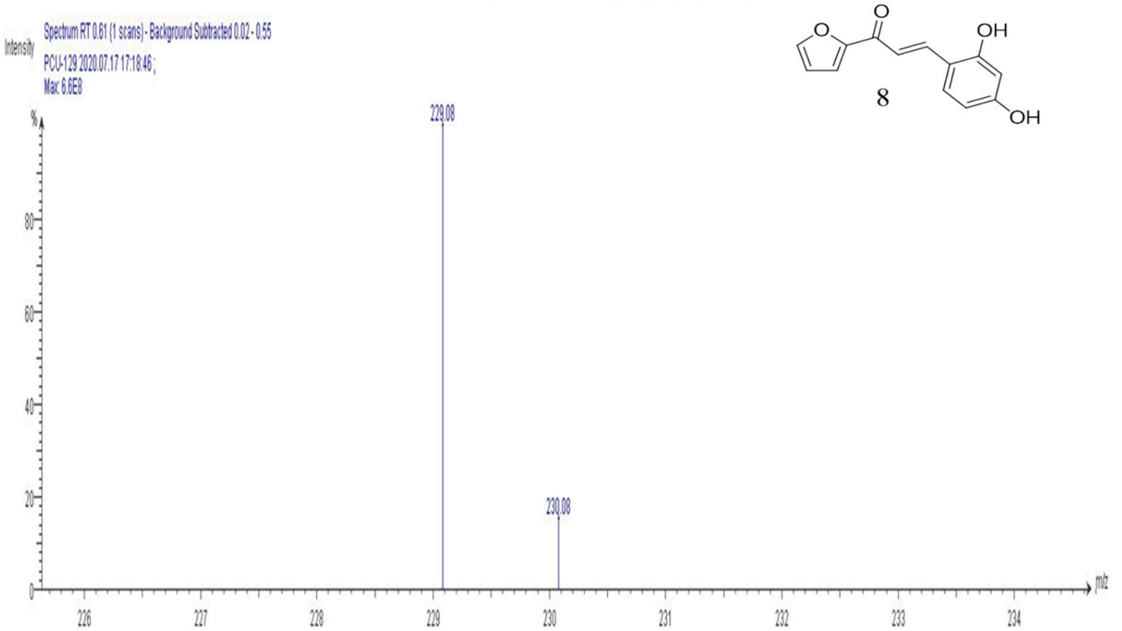

Fig. S24. ESI-MS spectrum of **8**

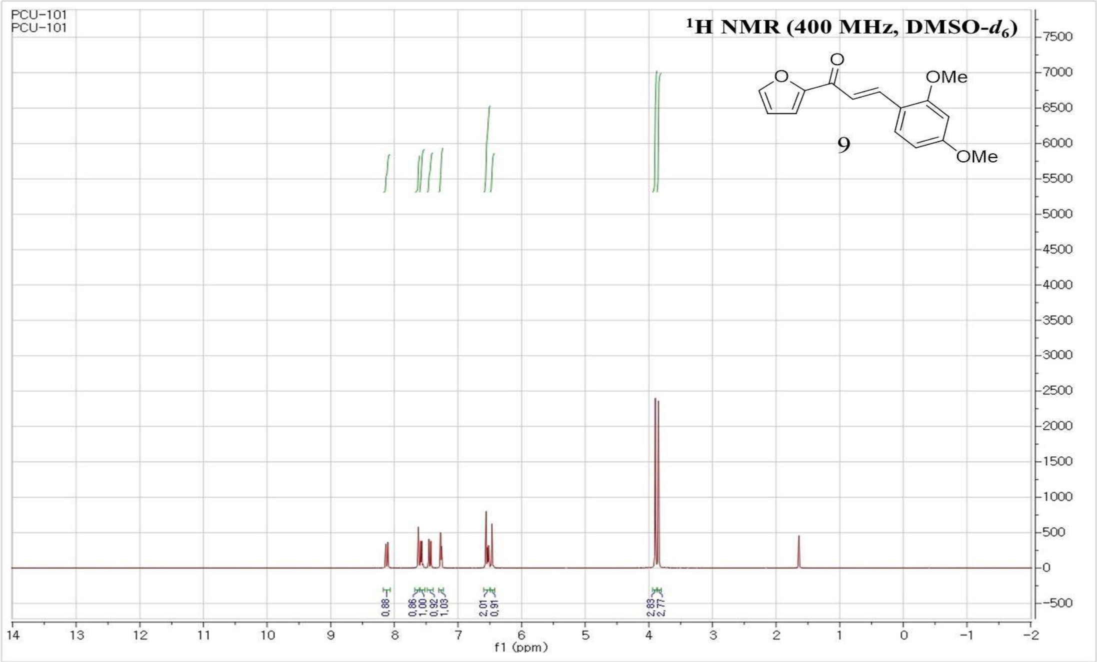

Fig. S25. ^{1}H-NMR spectrum of **9**.

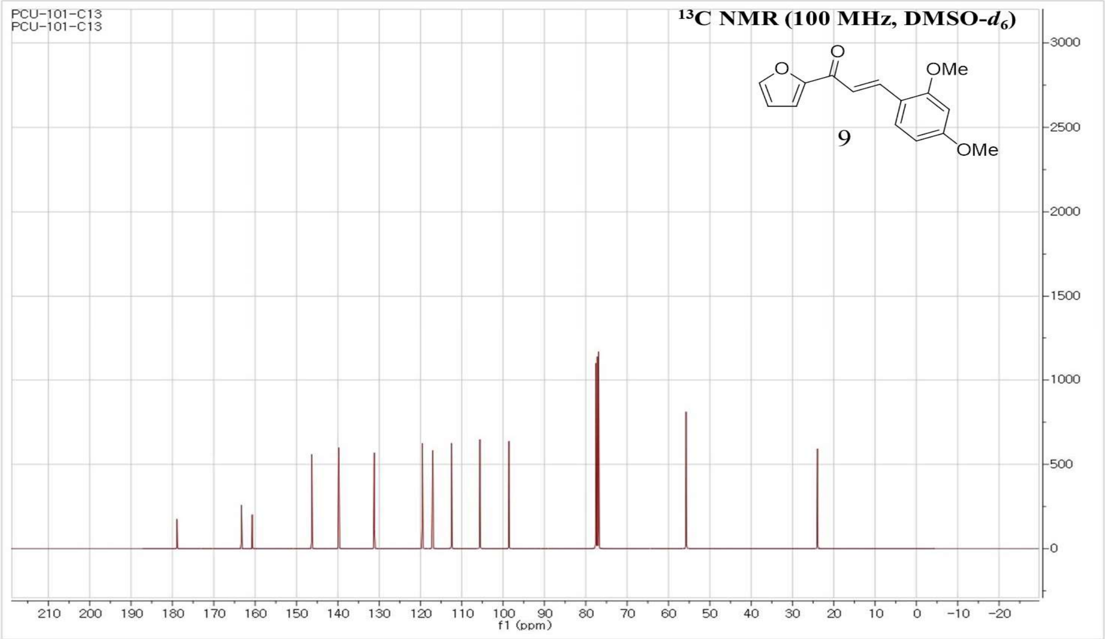

Fig. S26. ^{13}C-NMR spectrum of **9**.
